# Miscellanies

**Published:** 1857-02

**Authors:** 


### MISCELLANIES.

WE feel complimented, from time to time, in finding our sentiments, especially in reference to "Consumption," corroborated by leading minds in this and other countries—*or*, we corroborate theirs. In our book on this subject just published, page thirty-six, we make this statement:

"We know of no calling in human life which may not be pursued with impunity, which may not be pursued in such a way as to promote health, if done judiciously, wisely, moderately."

That excellent and substantial weekly—*Littell's Living Age*, No. 661—in an article from *Chambers' Edinburg Journal*, on the INFLUENE OF OCCUPATION ON HEALTH, says: "It is a mistake to think that the ill-health found in so many trades is a component part of them, or that those engaged in one occupation must necessarily be shorter lived, or suffer more, physically, than those of another. If we inquire closely into the matter, we shall find that every single instance of ill-health, arising from the different trades, may be fully accounted for by some breach of the simple laws of nature, and that the evils are capable of a remedy, so cheap and attainable, that it would be impossible for them to add appreciably to the expense of the article produced; so that, by *preventing* the sickness of the artisan, it would be the greatest saving to the proprietors, and to society at large."

There is also an article in No. 25 of the Knoxville *Journal of Medical and Physical Sciences*, among other good ones, by Dr. Frazier, recommending, as we have done in "Consumption," the mountains of East Tennessee as an admirable locality for consumptives, provided, when they get there, they will live in the open air. His illustrations are of great interest, and are highly encouraging to the invalid.

PRINCETON REVIEW for January, 1857. By Rev. Charles Hodge, D.D. This is by far the ablest Presbyterian periodical in any language, and is regarded by that Church as its orthodox exponent on all theological subjects. We say the same as to the "*Christian Review*" among the Baptists.

